# Health-Related Aspects of Milk Proteins

**Published:** 2016

**Authors:** Seyed Hossein Davoodi, Roghiyeh Shahbazi, Saeideh Esmaeili, Sara Sohrabvandi, AmirMohamamd Mortazavian, Sahar Jazayeri, Aghdas Taslimi

**Affiliations:** a *Cancer Research Center, Shahid Beheshti University of Medical Sciences, Tehran, Iran.*; b *Department of Clinical Nutrition and Dietetics, National Nutrition and Food Technology Research Institute, Faculty of Nutrition Sciences, Food Science and Technology, Shahid Beheshti University of Medical Sciences, Tehran, Iran. *; c *Students*^٫^* Research Committee, National Nutrition and Food Technology Research Institute, Faculty of Nutrition Sciences and Food Technology, Shahid Beheshti University of Medical Sciences, Tehran, Iran. *; d *Department of Food Technology Research, National Nutrition and Food Technology Research Institute, Students Research Committee, Shahid Beheshti University of Medical Sciences, Tehran, Iran.*; e *Department of Food Science and Technology, National Nutrition and Food Technology Research Institute, Faculty of Nutrition Sciences and Food Technology, Shahid Beheshti University of Medical Sciences, Tehran, Iran.*

**Keywords:** Whey, Casein, Peptide, Health, Nutrition

## Abstract

Milk is an important component of a balanced diet and contains numerous valuable constituents. Considerable acclaimed health benefits of milk are related to its proteins, not only for their nutritive value but also for their biological properties. Scientific evidence suggests that anticarcinogenic activities, antihypertensive properties, immune system modulation, and other metabolic features of milk, are affiliated with its proteins (intact proteins or its derivatives). In this article, the main health-related aspects of milk proteins, such as anticarcinogenic, immunomodulatory, antimicrobial, anticariogenic, antihypertensive, and hypocholesterolemic effects are reviewed. Collectively, the findings indicate the effectiveness of milk proteins on reduction of risk factors for cancer, cardiovascular diseases and overall improvement of health aspects.

## Introduction

Bovine milk is a liquid food (87% water) which contains an average of 13% total solids and about 9% solids-not-fat. Milk is a nutrient-dense food with important nutritional value due to its calcium, vitamin D (especially in fortified form), protein, vitamin B_12_, vitamin A, riboflavin, potassium, and phosphorus. Sufficient content of the amino acid tryptophan, a niacin precursor, highlights milk as an important source of niacin equivalents. Additionally, it contains different bioactive compounds with medicinal (nutraceutical) effects (1-4). Epidemiologic studies have manifested the association of milk and its products consumption in lower risk of metabolic disorders, cardiovascular diseases, hypertension, cancer, and some other diseases with (5-9). 

Total protein content of bovine milk is approximately 3.5% by weight (36 g/L), providing almost 38% of the total solids-not-fat content of milk, and about 21% of whole milk energy (4, 10). Milk is known as a major source of high-quality proteins that possesses a wide range of nutritional, functional, and physiological activities (11-12). Milk is also a unique source of peptides with biological activity. Peptides derived from casein fractions and whey proteins, including opioid peptides, antihypertensive peptides, casein phosphopeptides (CPPs), glycomacropeptide (GMP), and lactorphins, possess various physiological roles, such as opioid-like features, immunostimulating activities, anti-hypertensive activities, antibacterial and antiviral impacts and also enhancement of calcium absorption (13-18).The inovativity of this article is comprehensive review of the nutritional and therapeutic effects of milk proteins and peptides bioactivities which collects all the significant studies in the last 30 years and provides an update of current knowledge in one place.

*Milk proteins*

Casein and whey protein are the major proteins of milk. Casein constitutes approximately 80%(29.5 g/L) of the total protein in bovine milk, and whey protein accounts for about 20% (6.3 g/L) (19-21). Casein is chiefly phosphate-conjugated and mainly consists of calcium phosphate- micelle complexes (20). It is a heterogeneous family of 4 major components including alpha- (α_s1_- and α_s2_-casein), beta-, gamma-, and kappa-casein (2, 22, 23). 

Whey protein is a collection of globular proteins with a high level of α-helix structure and the acidic-basic and hydrophobic-hydrophilic amino acids are distributed in a fairly balanced form (24). Alpha-Lactalbumin (α-LA) and beta-lactoglobulin (β-LG) are the predominant whey proteins and comprise about 70–80% of the total whey proteins. Among the other types of whey proteins, immunoglobulins (Igs), serum albumin, lactoferrin (LF), lactoperoxidase (LP), and protease-peptones must be mentioned (19, 24-26). Whey proteins have substantial levels of secondary, tertiary, and quaternary structures. They are heat-labile stabilizing their prtotein structure through intermolecular disulfide linkages (25). 

*Nutritional benefits*

Bovine milk protein is considered a high-quality, or complete protein, because it contains all 9 of the essential amino acids in proportions resembling amino acid requirements (3-4). Due to the high quality of bovine milk protein, it is regarded as a standard reference protein to evaluate the nutritive value of other food proteins (4). Furthermore, branched-chain amino acid (isoleucine, leucine, and valine) contents in milk proteins are at higher levels than in many other food sources. These amino acids, especially leucine, help to minimize muscle wasting under conditions of increased protein breakdown and can stimulate muscle protein synthesis. Moreover, whey protein has a high content of sulfur-containing amino acids (cysteine and methionine) which are precursors of glutathione, a tripeptide with antioxidant, anticarcinogenic, and immunostimulatory properties (4, 28).

*Therapeutic benefits*

**Figure 1:**
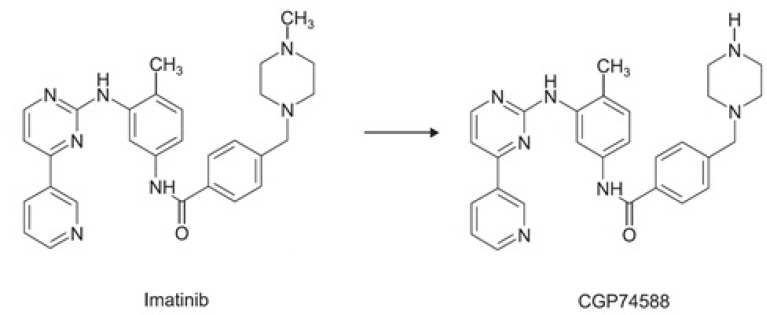
Molecular structure of imatinib and CGP74588

Caseins and whey proteins differ in their physiological and biological properties. In recent years, many studies have investigated the therapeutic aspects of milk proteins. These aspects of milk proteins are described below in Figure 1. Table 1 indicates selective publications on the health benefits of milk proteins.

*Therapeutic benefits of whey proteins*

*Anticarcinogenic effects*

Several studies suggest that milk proteins, especially whey proteins, may protect the human body against some cancers (colon, breast, and prostate gland) probably through their ability to enhance cellular levels of glutathione as well as promoting hormonal and cell-mediated immune responses (9, 29-34). It has been indicated that whey proteins such as LA, LG, LF, LP, and Igs possess anticarcinogenic activity (35). 

LF, an iron-binding glycoprotein from the transferrin family, has antiproliferative, anti-inflammatory, and antioxidant features (9, 36-40). Based on in vivo studies, oral administration of LF to rodents significantly decreased the chemically induced carcinogenesis in various organs such as breast, esophagus, tongue, lung, liver, colon, bladder, and hindered angiogenesis (37, 41, 42). However, the mechanisms of LF action is yet to be understood besides there are some evidences to support its capability to interact with some receptors and to modulate the genetic expression of several molecules which are vital to the cell cycle and apoptosis mechanisms (9).

The majority of findings suggesting the anticancer traits of whey proteins, have been acquired from in vitro studies using carcinoma cell lines or in vivo studies using animal models. In vitro studies examining chemically induced tumor formation have reported the inhibitory effect of whey protein supplementation on the incidence and growth of the tumors, as induced by 1,2-dimethylhydrazine (DMH) and azoxymethane (AOM), and might reduce the risk of developing colorectal cancer (43-45). Hakkak *et al*. (46) found that the incidence of mammary tumors induced by dimethylbenz-[α]-anthracene, a chemical substance used to produce mammary adenocarcinoma, was approximately 50% lower in female rats fed with 14% (w/w) whey protein compare to casein-fed rats, and approximately 30% less than soy-fed rats after 4 months. In another study by McIntosh et al. (47), rats on whey protein diet (20 g protein/100 g body weight) showed improved protection against dimethylhydrazine-induced intestinal tumors compared to animals fed an equal amount of soy protein or red meat. 

β-LG, as a rich source of cysteine, stimulates glutathione synthesis, an anticarcinogenic tripeptide produced by liver to protect against intestinal tumors (48). Moreover, in vitro growth inhibition of MCF-7 human breast cancer cell by bovine serum albumin (BSA) has been reported (49). Also, bovine α-LA, in a concentration of 5 to 35 microg/mL, exerted an antiproliferative and apoptotic activity against some types of human colon cancer cell lines such as Caco2 and Ht-29 (50). 

*Immunomodulatory effects*

Various *in vitro* and *in vivo* studies have proven that milk whey proteins are able to positively influence immune responses. Mice fed with whey protein concentrate (for 12 weeks) showed significantly higher mucosal antibody responses to ovalbumin and cholera toxin compared to those fed a normal diet (51). 

Ingestion of bovine whey proteins (for 5 to 8 weeks) was recognized to improve footpad delayed-type hypersensitivity responses and *in-vitro* concanavalin A-induced spleen cell proliferation in mice (52). The influence of whey protein concentrate on T-cell populations has also been reported. Mice fed with 25 g undenatured whey protein concentrate (for 4 weeks) exhibited higher numbers of L3T4+ cells (helper cells) and a higher ratio of L3T4+/Lyt-2+ cells (helper/suppressor) compared to those fed an isocaloric casein diet (53). A significant increase in total white blood cells, CD4+ and CD8+ lymphocyte counts, and concanavalin A-stimulated interferon-gamma (IFN-γ) production by spleen cells has also been observed in alpha whey fraction-fed mice compared to mice fed with casein and soy protein isolate(54).

One study announced a dose-dependent improvement of delayed-type hypersensitivity responses to a range of antigens, including ovalbumin, sheep red blood cells, and *Calmette- Guerin bacillus* in mice, after oral or parenteral administration of bovine LF (55). 

An *in-vitro* study reported that modified whey protein concentrate (mWPC) suppressed T and B lymphocyte proliferative responses to mitogens in a dose-dependent manner*,* while it also suppressed alloantigen-induced lymphocyte proliferation during a mixed leukocyte reaction. Additionally, cytokine secretions, IFN-γ and interleukin-4 (IL-4), and the percentage of activated CD25+ T cell blasts following mitogen stimulation, were suppressed by the mWPC (56). It has been observed that oral administration of bovine LF promoted antimetastatic activity and strongly increased the numbers of CD4+, CD8 +, and natural killer (NK) cells in the lymphoid tissues, small intestine, and peripheral blood of tumor-bearing mice. Moreover, it enhanced the cytotoxic activities of these cells against Yac-1 lymphoma cell and colon 26 carcinoma. In addition, it significantly augments production of IL-18, IFN-γ, and caspase-1 in the small intestine (37, 57).

In cancer patients, prescription of whey protein (30 g daily for 6 months) has been demonstrated to normalize the number of blood leukocytes (58). Also, supplementation with whey protein has been reported to increase plasma glutathione levels and natural killer (NK) cell activity in patients with chronic hepatitis B (59). 

*Antimicrobial and antiviral effects*

Intact whey contains a number of unique components with broad antimicrobial activity. Several studies have demonstrated the inhibitory activity of whey proteins against *Helicobacter pylori* (*H*.* pylori*) in infected subjects. In a study in fifty-nine healthy subjects, Okuda *et al*.(60) revealed that twice daily oral administration of LF tablets (200 mg) for 12 weeks decreased the ability of *H. pylori *to form colonies*,* but complete eradication was not achieved. In a large multicentered trial, the eradication rate of *H. pylori* in the infected patients receiving LF (200 mg) twice a day for 7 days was 73% (61). 

LF has been shown to render direct bactericidal activity against Gram-negative organisms due to its ability to bind to the lipid A part of bacterial lipopolysaccharides and to increase membrane permeability (62). It was found that LF (1 mg/mL) significantly protected cultured epithelial cells (isolated from patients suffering from pharyngitis) against *in vitro* invasion by group A *Streptococcus (*GAS) and intensely prevented invasiveness of GAS pretreated by erythromycin or ampicillin(63). The efficacy of bovine milk Ig concentrate against *Shigella flexneri* and protection against shigellosis among healthy adult subjects has been reported by Tacket *et al*. (64). Furthermore, bovine milk-derived Igs could protect against oral challenge with enterotoxigenic *Escherichia coli(E. coil)*in healthy adult volunteers(65). A significant reduction in growth and cell numbers of an infant fecal microorganism, *E. coil* 2348/69, in infants fed with a formula supplemented with α-LA was reported by Brücket al. (66). 

Moreover, some studies showed antiviral activity of whey proteins. Some research has examined the inhibitory activity of whey proteins against human immunodeficiency virus (HIV). LF, α-LA, and β-LG have shown inhibitory activities against HIV-1. LF exhibited strong inhibitory activity against HIV-1 reverse transcriptase activity, but weak inhibitory activity against HIV-1 protease and integrase, while α-LA and β-LG exerted inhibitory activity against HIV-1 protease and integrase but did not inhibit HIV-1 reverse transcriptase. LF was more effective during the early stage of HIV infection (67-68).

*Anticariogenic effects*

There is much scientific evidence that supports protective impacts of whey proteins against dental caries. It has been indicated that whey might have a topical anticariogenic impact by its buffering capacity (69). Mitoma *et al*. (70) demonstrated that bovine LF can be firmly bound to salivary agglutinin and therefore inhibit the interaction between protein antigen of *Streptococcus mutans *(*S. mutans*) and salivary agglutinin. In another study, inhibition of *S. mutans* adherence to saliva-coated hydroxyapatite (S-HA) by milk components was demonstrated. Bovine LF showed the strongest inhibitory activity. Other components, such as LP and IgG revealed moderate inhibitory activities (71). Also, LP and lysozyme synergistically provided anticariogenic effects through restricting glucose metabolism by *S. mutans* and therefore reduced acid production in the dental plaque environment (25, 71). 

*The impact of whey proteins on satiety, food intake, and weight loss*

The effects of milk and milk products on the regulation of food intake and satiety have been attributed to several components. Among milk components, proteins possess the greatest potential to provide satiety signals and milk proteins are more satiating than other protein sources (72-74). Whey proteins contribute to the short-term and long-term food intake regulation by inducing satiety signals (75-76). One study showed that consumption of 45 g whey protein, in the form of sweetened beverages, suppressed food intake more than egg albumin and soy protein at a pizza meal 60 min later (77). In another study, drinks containing 400 Kcal and 48 g of whey stimulated subjective satiety, and reduced food intake at a buffet meal 90 min later, more than the drinks containing the same amount of casein(78). A high-protein breakfast (58.1% of energy from protein and 14.1% of energy from carbohydrate) consisting of dairy products enriched with whey protein isolate raised glucagon-like peptide-1 levels over 3 h more than a high-carbohydrate breakfast (19.3% of energy from protein and 47.3% of energy from carbohydrate) consisting of plain yogurt (79). 

In a clinical trial with healthy overweight and obese participants,Baer *et al*.(80) found that after 23 weeks of consumption of supplemental whey protein, soy protein, and an isoenergetic amount of carbohydrate, body weight and body fat among the whey protein group were lower than the group consuming carbohydrate. Waist circumference was also smaller in the subjects receiving whey protein than in the other groups. Moreover, fasting ghrelin was lower in subjects receiving whey protein in comparison with soy protein or carbohydrate.

Feeding insulin-resistant obese rats with whey protein has been shown to reduce calorie intake, to decrease body fat, and therefore to result in a significant improvement in insulin sensitivity in comparison with a red meat diet (81-82). Furthermore, in rats receiving high-protein diets ad libitum over a 25-day period, milk protein fractions (whole milk protein, whey protein, or β-LG-enriched fraction) reduced calorie intake, body weight, and body fat. β-LGwas the most efficient fraction (83).

*Therapeutic benefits of casein proteins*

*Anticarcinogenic effects*

Evidence indicates that casein might protect the body against some cancers. Casein inhibits fecal beta-glucuronidase, an enzyme produced by intestinal bacteria and deconjugates procarcinogenic glucuronides to carcinogens (21). Casein might also protect against colon cancer through its influence on the immune system, specifically by stimulating phagocytic activities and increasing lymphocytes (29). Other researchers suggest that the anticarcinogenic properties of casein are associated with its molecular structure (84). 

Lower incidence of DMH-induced colorectal cancer was found in rats fed a casein diet compared to rats fed other sources of protein such as soybean and red meat. The intracellular concentration of glutathione in the liver was also greater in the casein-fed rats (47). A reduced incidence of colon tumors was also observed in rats fed a mixture of casein and wheat protein compared to those fed with the equivalent amount of wheat and chickpea protein (85). In an investigation, rats treated with 10 weekly injections of 7.4 mg/Kg body weight of AOM, received synthetic isoenergetic diets with different amount of protein content including 25% casein (normal-protein diet), 10% casein (low-protein diet), or 5% casein (very-low-protein diet). Administration of a diet containing 25% casein resulted in a fewer incidence of colon tumors in rats than isoenergetic diets containing 10 and 5% casein after 30 weeks (86). 

In-vitro and in-vivo studies have demonstrated the impact of caseinate and soy protein on the mutagenic potential of N-methyl-N′-nitro-N-nitrosoguanidine (MNNG). Of these 2 dietary proteins, only casein presented antimutagenic activity against MNNG in the small intestine of mice treated with this mutagen (87). In addition, the antimutagenic potential of casein was assessed against different mutagens, including some food-related mutagens. Casein showed the most antimutagenic activity against benzo[a]pyrene, N-methylnitrosourea, and nitrosated 4-chloroindole, and the least antimutagenic activity against sodium azide and N-nitroquinoline-1-oxide(88). 

*Anticariogenic effects*

Some studies indicate that casein might contribute to the beneficial effects of milk on oral health(89). Kappa-casein (k-casein) may protect against dental caries by decreasing the activity of glucosyltransferase, a plaque-promoting enzyme produced by *S. mutans*, and the ability of this enzyme to adhere to dental surfaces or S-HA (90). -Casein has also been revealed to reduce the adherence of *S. mutans* to the S-HA surfaces of teeth (91-92). 

A study in rats infected by mixed bacterial suspensions of *Streptococcus sobrinus OMZ 176 *and *Actinomyces viscosus Ny1*indicated that consumption of powdered milk micellar casein could reduce the formation of advanced dental fissure and smooth surface lesions, and inhibit colonization of *Streptococcus sobrinus (S. sobrinus) *(93). In another study whole casein was combined with a citric acid solution in order to assaying the impact of soft drinks on the hydroxyapatite dissolution rate. Adding 0.02% (w/v) casein to citric acid solutions significantly decreased the hydroxyapatite dissolution rate by approximately 50–60% (94). 

*Hypocholesterolemic effects*

Some investigators have studied the effect of casein on blood cholesterol. In a crossover study, 11 normal participants received diets providing 20% of calories from casein or soy protein. The mean of cholesterol intake was 500 mg/d. An initial reduction in plasma cholesterol and low-density lipoprotein cholesterol (LDL-C) was observed in both diets (95). In another crossover study, normolipidemic nonobese healthy men consumed 2 liquid-formula diets containing casein or soy protein. After 30 days on each diet, the lipoprotein (a) concentration was reduced by approximately 50% with the casein dietcompared to the soy-protein diet. Total cholesterol, LDL- C, and high-density lipoprotein cholesterol (HDL-C) concentrations also were lowered with both diets(96). In hypercholesterolemic subjects who consumed 2 doses of casein (30 or 50 g) in the form of beverage, total cholesterol concentrations were reduced during 16 weeks (97). One study in Australian individuals at high risk of developing heart disease showed daily supplementation with 25 g of beta-casein* (*β-casein) A1 or A2 could significantly reduce blood cholesterol concentrations (98). 

*Therapeutic benefits of bioactive peptides derived from milk proteins*

Milk contains different bioactive components, including bioactive peptides with physiological functionality. Peptides generated from milk include a variety of substances which are potent modulators of various regulatory processes in the body and exhibit multifunctional bioactivities. Biologically active peptides hidden within the intact milk proteins are released and activated by gastrointestinal digestion of milk, fermentation of milk by proteolysis starter cultures, or hydrolysis by proteolytic enzymes. Bioactive peptides derived from casein and whey proteins, including opioid peptides, antihypertensive peptides, CPP, lactorphins, and albutensin have been demonstrated to play physiological roles such as opioid-like features, immunostimulation, angiotensin­ I-converting enzyme (ACE) inhibition, anti-hypertensive property, and antimicrobial activity (13, 14, 99-105). 

*Therapeutic benefits of bioactive peptides derived from whey*

Hydrolysis of whey proteins generates bioactive peptides .Experimental findings have revealed that bioactive peptides can be purified from α-LA, β-LG, bovine LF, and BSA. Some of these peptides have been given particular names such as α- and β-lactorphin, β-lactotensin, serophin, albutensin A, lactoferricin (Lfcin), and lactoferrampin. Most of these peptides have not been characterized to the extent of casein-derived peptides.Recently, whey-derived peptides have received special attention, because of their preventive and therapeutic characteristics (14, 106, 107). Different therapeutic benefits of whey-derived bioactive peptides are discussed below.

*Anticarcinogenic effects*

Peptides derived from the N-terminal region of LF have been investigated in order to identify sequences with potential antitumor activity. Roy *et al*.(108) isolated 4 peptides from pepsin hydrolysates of lactoferrin with antiproliferative and apoptotic property. The sequence corresponding to residues 17–38 of bovine LF showed the highest apoptotic activity in human leukemia cells (HL-60). Eliassen et al.(109) reported that bovine Lfcin, f (17-41), exhibitedcytotoxic activity against Meth Afibrosarcoma, melanoma, and colon carcinoma cell lines, and significantly lowered the size of solid Meth A tumors. Also, Lfcin displayed antitumor activity against MDA-MB-435 breast cancer cells by inducing apoptosis (110) and cytotoxic activity *in-vitro* and *in-vivo* against neuroblastoma cells by destabilization of the cytoplasmic and the mitochondria membranes(111). 

Lfcin B could also inhibit angiogenesis mediated by vascular endothelial growth factor and fibroblast growth factor in mouse models, as well as to mediate antiproliferative andantimigratory activities against proangiogenic factor-induced human umbilical vein endothelial cells (112). 

*In-vitro* studies suggest treatment with Lfcin B induced apoptotic death in several different human leukemia and carcinoma cell lines by stimulating the mitochondrial pathway of apoptosis via the production of reactive oxygen species and activation of caspase-9 and caspase-3(113). In addition, it has been observed that bovine Lfcin can trigger mitochondrial-dependent apoptosis in Jurkat T-leukemia cells by cell membrane damage through binding to the cell membrane, increasing permeabilization of the cell membran, and the subsequent disruption of the mitochondrial membrane(114).

*Immunomodulatory effects*

Whey includes several potent immunomodulatory peptides that are hidden within the intact structure of whey proteins (115). The impact of peptides liberated by tryptic digestion of bovine β-LG on various immune functions in mice was studied by Pecquet *et al*. (116). The tolerance to β-LG was enhanced in mice fed β-LG hydrolysates or fractions of the hydrolysate. A reduction in serum and intestinal IgE levels was also observed. Furthermore, β-LG-specific delayed-type hypersensitivity and proliferative responses of splenic cells were suppressed.

Prioult *et al*.(117) announced that hydrolaysate of β-LG with *Lactobacillus paracasei* peptidases generated a number of small immunomodulatory peptides. These peptide sequences reduced lymphocyte proliferation and enhanced immunosuppressant interleukin-10 production.

Several studies have revealed the immunomodulary properties of Lfcin. Hydrolysis of bovine LF with pepsin produced some immunostimulatory and immunoinhibitory peptides. Thehydrolysate significantly enhanced proliferation and Igs (IgM, IgG, and IgA) generation in murine splenocytes and also proliferation and IgA production in Peyer’s patch cells *in-virto*(118). Bovine LF and Lfcin B were found to reduce the IL-6 response in THP-1 human monocytic cellsafter stimulating by lipopolysaccharide(119). In addition, Lfcin B augmented the phagocytic activity of human neutrophils through direct binding to the neutrophils and opsonin-like activity (120). 

*Antimicrobial effects*

LF-derived peptides have been identified to present antimicrobial properties. The antibacterial features of enzymatic hydrolysates of bovine LF were investigated by Tomita *et al*.(121). Hydrolysates prepared with cleavage of LF by porcine pepsin, cod pepsin, or acid protease from *Penicillium duponti*exerted intense antibacterial activity against *Escherichia coli* 0111. 

It was shown that Lfcin B inhibited or inactivated various ranges of Gram-positive and Gram-negative bacteria, including *E. coli*, *Salmonella enteritidis*, *Yersinia enterocolitica, Klebsiella pneumoniae*, *Proteus vulgaris*, *Pseudomonas aeruginosa*, *Campylobacter jejuni*, *Staphylococcus aureus*,*Staphylococcus haemolyticus*, *Streptococcus thermophilus*,*S. mutans*, *Clostridium perfringens, Corynebacterium diphtheriae*, *Listeria monocytogenes*, *Bacillus subtilis (B. subtilis )*, and *Bifidobacterium infantis*(122-124). 

Proteolytic cleavage of α-LA generated 3 antibacterial peptide fragments including LDT1 f(1–5), LDT2 f(17–31SS109– 114), and LDC f(61–68S-S75–80). These sequences were effective against Gram-positive bacteria, while they presented weak activity against Gram-negative bacteria (124).Furthermore, 4 peptide fragments including f(15–20), f(25–40), f(78–83), and f(92–100), were isolated by tryptic digestion of bovine β-LG. Releasedfragments revealed bactericidal activity against Gram-positive bacteria (126). 

*Antihypertensive effects*

It has been recognized that *in-vitro *incubation of milk proteins with gastrointestinal protease, including pepsin, trypsin, and chymotrypsin, can yield a large number of fragments with ACE inhibitory activity. The ACE inhibitory peptides are produced during gastrointestinal transport. Bacterial and plant proteinases can be applied to produce such peptides as well (127-128).

Nurminen *et al*. (129) examined the antihypertensive activity of alpha-lactorphin, a tetrapeptide (Tyr-Gly-Leu-Phe) originating from milk α-LA, in conscious spontaneously hypertensive rats (SHR) and in normotensive Wistar Kyoto rats. α-Lactorphin lowered blood pressure in SHR and Wistar Kyoto rats dose-dependently. Enzymatic cleavage of the whey protein by proteinase K released 6 potent ACE inhibitory peptides. These peptides possessed antihypertensive activity in SHR. Of these 6 peptides, the fragment Ile-Pro-Ala, originally derived from β-LG, exhibited the most ACE inhibitory feature (130). 

Mullally *et al*. (131) investigated the ACE inhibitory activity of a tryptic cleavage of bovine β-LG. The β-LG fraction (142–148) gave an ACE inhibition index of 84.3%. In another investigation, some ACE-inhibitory peptides were isolated by hydrolysis of bovine whey proteins with an enzyme combination, including pepsin, trypsin, and chymotrypsin, or with trypsin alone. The generated peptides were α-LA fragments (50-52), (99-108), (104-108), and β-LG fragments (22-25), (32-40), (81-83), (94-100), (106-111), (142-146). ACE activity was 50% suppressed by the whey hydrolysates at the concentration ranges of 345-1733 µg/mL (132). 

In addition, enzymatic digestions of LF released some antihypertensive peptides with molecular masses lower than 3 kDa. These fractions showed inhibitory activity against ACE and endothelin-converting enzyme (ECE) *in-vitro *(133).

Ruiz-Giménez et al.(134) reported that a set of 8 LfcinB (20-25)-generated peptides could inhibit ACE activity *in-vitro*. Of these peptides, 7 exerted *ex-vivo* inhibitory activity against ACE-dependent vasoconstriction. Only Oral administration of LfcinB (20-25) and one of its fragments, F1, reduced blood pressure in SHR.

Moreover, in a controlled study with prehypertensive or stage 1 prehypertensive human volunteers, blood pressure was significantly lower in the treatment group that consumed 20 g/day hydrolyzed whey protein isolate rich in bioactive peptides than in the control group that consumed the same amount of unhydrolyzed whey protein isolate(135).

*Therapeutic benefits of bioactive peptides derived from casein*

Casein, in either milk or dairy foods, is a main source of bioactive peptides. Casein-derived peptides reveal different bioactive roles (14). Below, the therapeutic advantages of casein-derived bioactive peptides are discussed.

*Anticarcinogenic effects*

According to various cytochemical studies, there is some evidence for the possible anticarcinogenic activity of casein-derived peptides. *In-vitro* examinations have indicated that casein-based peptides isolated after microbial fermentation of milk could protect against colon cancer through changing cell kinetics (84). Kampa *et al*.(136) described thatseveral casomorphin peptides, a group of opioid peptides derived from ɑ- and β-casein, suppressed the proliferation of some prostatic cancer cell lines, including LNCaP, PC3, and DU145, via involving opioid receptors. Also, apoptosis of HL-60 cells was promoted by the opioid peptide β-casomorphin-7 and the phosphopeptide β-CN (f1-25)4P (137). Moreover, purified peptides, corresponding to bioactive fractions of casein, showed modulatory effects on cell viability, proliferation, and apoptosis in various human cell culture models, including human peripheral blood lymphocytes, HL-60, polymorphonuclear leukocytes, and Caco-2 cells(138-139). 

*Immunomodulatory effects*

Some experiments have been conducted to consider the effect of casein-derived bioactive peptides on immune function. It was found that *in-vitro* digests of casein produced by peptidases of *Lactobacillus rhamnosus* inhibited protein kinase C translocation and downregulated IL-2 mRNA expression. These findings demonstrated *in-vitro* suppression of T cell activation by casein digests (140). Sütaset al.(141) reported that bovine caseins hydrolyzed with enzymes produced by*LactobacillusGG*inhibitedIL-4 production of peripheral blood mononuclear cells in atopic children. In another study, Sütas *et al*.(142) showed that digestion of caseins with proteasesgenerated from *Lactobacillus casei (L. casei) GG*,produced some fractions with suppressive effects on lymphocyte proliferation *in-vitro*. Hata *et al*.(143) demonstrated that caseinophosphopeptides β-CN(f1–25)4P and α_S1_-CN(f59–79)5P possessed immunostimulatory effects via increasing IgG production in mouse spleen cell cultures. Moreover, CPPs derived from bovine αs2- and β-casein exerted immuno-enhancing activity by enhancing the level of serum and intestinal antigen-specific IgA in mice fed with a CPPs preparation (144). 

Bovine GMP can stimulate human monocytes and secretion of tumor necrosis factor, IL-1β and IL-8 from human monocytes, through affects on mitogen-activated protein kinase and nuclear factor-kappaB signaling pathways. GMP might have an indirect intestinal anti-inflammatory impact through enhancing host defenses against invading microorganisms (145). GMP and its derivatives generated by peptic hydrolysis can stimulate proliferation and phagocytic activities of the human U937 macrophage-like cells (146). 

*Antimicrobial and antiviral effects*

There is some evidence regarding antimicrobial properties of casein-derived peptides. McCann *et al*. (147) discovered a novel fragment from bovine α_S1_-casein, f(99-109), purified by enzymatic digestionof bovine sodium caseinate with pepsin. This fragment exhibited inhibitory activity against Gram-positive and Gram-negative bacteria. 

Kappacin, the monophosphorylated fragment Ser(P)149 k -casein -A f(138 -158), produced by endoproteinase Glu-C digestion of CPP, displayed inhibitory activity against *S. mutans, Porphyromonas gingivalis*, and *E. coli*(148). Caseicidin, a defense peptide purified by chymosin hydrolysis of casein at neutral pH, showed inhibitory activity against *staphylococci*, *Sarcina spp*, *B. subtilis*, *Diplococcus pneumoniae*, and *Streptoco ccus pyogenes*(149). The immunomodulatory peptide isolated from bovine β-casein, β-CN (193-209) peptide, promotes the antimicrobial activity of mouse macrophages via up-regulation of MHC class II antigen expression and enhancement of phagocytic activity (150). The antimicrobial property of caseicins has been well demonstrated. Caseicins A and B, corresponding to f(21-29) and f(30-38) of bovine α_s1_-casein, showed an intense activity against *Enterobacter sakazakii*(151). 

*In-vitro* studies have revealed that casocidin-I, a C-terminal fragment of bovine α_S2_-casein, inhibited the growth of *E. coli* and *Staphylococcus* strains (152).Antibacterial and antiviral features of GMP have also been demonstrated. The ability of GMP to inhibit binding of cholera toxin to normal Chinese hamster ovary cells was reported by Kawasaki *et al*. (153). Furthermore, it showed similar inhibitory activity against *E. coli* heat-labile enterotoxins LT-I and LT-II, associated with colonization factor antigen CFA/I and CFA/II, respectively, in the Chinese hamster ovary model (154). GMP could also inhibit hemagglutination by 4 strains of human influenza virus at concentration ranges from 10^-2^ to 10^-^3 (155). Dosako *et al*. (156) demonstrated the ability of GMP to inhibit the morphological transformations in peripheral blood lymphocytes induced by *Epstein Barr* virus.

*Anticariogenic effects*

Some researchers have assayed the ability of casein’s bioactive peptides to inhibit demineralization and to enhance remineralization of tooth enamel. Milk-derived bioactive peptides such as CPP and GMP may be responsible for the cariostatic properties of cheese via suppressing the growth of cariogenic bacteria, concentrating calcium and phosphate in plaque, reducing enamel demineralization, and enhancing remineralization (25, 89, 157). 

The anticariogenic impacts of CPPs have been demonstrated in animal and human experiments. It was suggested that CPPs stabilized calcium phosphate by forming casein phosphopeptide-calcium phosphate complexes (CPP-CP) and increasing the uptake of calcium and phosphate by dental plaque (158-159). In addition, CPP and amorphous calcium phosphate (ACP) bind to plaque, providing a potential source of calcium within the plaque and decreasing the diffusion of free calcium. Therefore, CPP-ACP can protect against dental caries by reducing mineral loss during a cariogenic episode and supplying a rich source of calcium for subsequent remineralization (160-161). Additionally, CPPs might exert an anticariogenic impact by competing with plaque-forming bacteria for binding to calcium(162). 

Neeser *et al*. (163) investigated the ability of milk casein components to restrict the adhesion ability of some odontopathogenic bacteria to tooth surface. Sodium caseinate, CPP, and GMP inhibited adherence of potential dental pathogens, including *Streptococcus sobrinusOMZ 176 *as well as *Streptococcus sanguis* (*S. sanguis)OMZ* to S-HA beads. In a similar study, Schüpbach *et al*. (164) considered GMP and CPP as adhesion inhibitors of oral bacteria. Adhesion ability of *S. sobrinus* to salivary pellicle was decreased 49%, 75%, and 81% by GMP, CPP, and the combination of GMP and CPP, respectively.

*Antihypertensive effects*

Considerable research has been performed to investigate the impact of biologically active peptides obtained from casein on blood pressure. In a single-blind, placebo-controlled study with Japanese adults having high-normal blood pressure or mild hypertension, receiving a casein hydrolysate containing bioactive peptides (for 6 weeks), led to significant reduction in systolic blood pressure from 1.7 to 10.1 mm Hg, in a dose-dependent fashion (165). A study in normotensive and mildly hypertensive patients showed consumption of 10 gr of a tryptic digest of casein (twice daily for a 4-week period) had an antihypertensive influence (166). In another investigation, daily ingestion of 800 mg/Kg body weight of a casein hydrolysate product for 6 weeks decreased the development of hypertension and increased the eNOS expressionin SHRs (167). 

In a placebo-controlled study, daily consumption of 95 mL sour milk containing two ACE-inhibitory peptides from β-casein, f(84–86) and f(74–76), significantly attenuated the blood pressure of hypertensive participants after 4–8 weeks (168). It has been reported that casein-derived peptides by *L. helveticus* proteases indicated ACE inhibitory activities (169). ACE inhibitory activity of the casein-based tripeptides Ile-Pro-Pro and Val-Pro-Pro has also been revealed by Nakamura *et al*. (170).

In a placebo-controlled double-blind crossover study, consumption of a product containing casein-derived tripeptides (Ile-Pro-Pro and Val-Pro-Pro) and plant sterols acutely reduced blood pressure in individuals with mild hypertention (171). 

**Table 1:** Selected publications on health benefits of milk proteins

**Type of protein**	**Biological function**	**Note**	**Reference**
Whey proteins Whey protein concentrate	Anticarcinogenic activity	Inhibition of incidence and growth of chemically induced tumors	43, 44, 45
	Immunomodulation	Higher mucosal antibody responses to antigens	51
		Impact on T-cell populations, increase in the T-helper cells concentration and T-helper cells/T-suppressor cells ratio	53
β-Lactoglobulin	Anticarcinogenic activity	Stimulation of the glutathione synthesis	48
	Antiviral activity	Inhibition of *HIV-1 *protease and integrase activities	67
			
α-Lactalbumin	Anticarcinogenic activity	Antiproliferative action on colon adenocarcinoma cell lines	50
	Antibacterial and antiviral activity	Reduction in cell numbers of the infant fecal *E. coli*	66
		Inhibition of *HIV-1* protease and integrase activities	67
Lactoferrin	Anticarcinogenic activity	Antiproliferative, anti-inflammatory and antioxidant activities	9, 36- 40
	Immunomodulatin	improving delayed-type hypersensitivity responses to a range of antigens	55
		antimetastatic activity and increase in the numbers of CD4+, CD8+, and NK cells in mice	59
	Antibacterial activity and antiviral activity	Inhibitory effect against *H.* *pylori*	60, 61
			
		Antibacterial activity against Gram-negative organisms	62
		Inhibition of *HIV-1* reverse transcriptase, protease and integrase activities	67, 68
	Anticariogenic activity	Inhibition of the interaction between *S. mutans* and salivary agglutinin	70
		Inhibition of *S. mutans *adherence to S-HA	71
Immunoglobulin	Antibacterial activity	Prevention of shigellosis	64
		Protection against oral challenge with enterotoxigenic *E.coli*	65
	Anticariogenic activity	Slight inhibitory activity against *S. mutans* adherence to S-HA	71
Casein Whole casein	Anticarcinogenic activity	Protect against colon cancer	85
		Decreasing the incidence of chemically induced intestinal tumors	47, 86
		Antimutagenic effect in the small intestine	87, 88
	Anticariogenic activity	Reduction in the hydroxyapatite dissolution rate	94
	Hypocholesterolemic effects	Reduction in the total cholesterol, LDL-C, HDL-C and lipoprotein (a) concentrations	95, 96, 97
k-Casein	Anticariogenic activity	Reduction in the activity of the plaque-promoting enzyme	90
		Inhibiting the adherence of *S. mutans* to the S-HA surfaces of teeth	91, 92
β-Casein	Hypocholesterolemic effects	Reduction in blood cholesterol levels	98
Bioactive peptides Lactoferricin	Anticarcinogenic Activity	Cytotoxic, antitumor, and apoptotic activity against cancer cell lines	109, 110, 111
		Inhibition of tumor angiogenesis mediated by growth factors in mice	112
	Immunomodulation	Increase in Igs (IgM, IgG, and IgA) production	118
		Decrease in the IL-6 response in a monocytic cell line	119
		Increasing the phagocytic activity of human neutrophils	120
	Antibacterial activity	Growth inhibition of diverse range of Gram-positive and Gram-negative bacteria	122, 123
			
	Antihypertensive activity	Inhibition of ACE activity and ACE-dependent vasoconstriction	134
Lactorphin	Antihypertensive activity	Decrease in blood pressure in hypertensive rats	129
Casein- phosphopeptides	Anticariogenic activity	Stabilization of calcium phosphate, decreasing the mineral loss during cariogenic episode	158, 160, 161
		Inhibition of *S. sobrinus *and *S. sanguis* adherence to S-HA	163
Kappacin	Antibacterial activity	Inhibition of* S. mutans, Porphyromonas* gingivalis and *E. coli*	148
Caseicidin	Antibacterial activity	Antibacterial activity against *staphylococci, sarcina, B. subtilis, Diplococcus pneumoniae and Streptococcus pyogenes*	149
Caseicins	Antibacterial activity	Inhibitory activity against *Enterobacter sakazakii*	151
Glycomacropeptide	Antiviral activity	Inhibition of against human *influenza* virus and* Epstein Barr* virus	155, 156
	Immunomodulation	Indirect anti-inflammatory effect of intestinal by Promotion host defense against microorganisms	145
		Enhancing of proliferation and phagocytic activities of human macrophage-like cells	146
Casomorphin peptides	Anticarcinogenic activity	Decrease in proliferation of prostatic cancer cell lines	136
		Promotion of apoptosis in human leukemia cells (HL-60)	137

## Conclusion

Milk is the oldest and one of the most widely consumed nutritious foods worldwide. It is highlighted as a source of high-quality proteins and one of the most important sources of bioactive peptides. Milk proteins have high nutritive value and remarkable medicinal properties. They are known as potential ingredients of health-promoting functional foods, and the dairy industry has already commercialized many milk proteins and peptide-based products which can be consumed as part of a regular daily diet. They are consumed by infants, the elderly, and immune-compromised people. They are also consumed to maintain good health status and prevent diet-related chronic diseasessuch as obesity, cardiovascular disease, and cancer. Milk-derived peptides are commonly ingested both in functional foods and drugs. They exhibit various well-defined pharmacological effects, for example, in the treatment of diarrhea (casomorphins), hypertension (casokinins), thrombosis (casoplatelins), dental diseases, mineral malabsorption (CPPs), and immunodeficiency (immunopeptides). These findings introduce new perspectives in the nutritional and technological evaluation of milk products and encourage utilization of these substances for production of food and new health promoting products. More studies related to the mechanisms by which these proteins exert their effects are required to achieve further substantial evidence. 

*Conflict of interest*

The authors confirm that this article content has no conflicts of interest.
